# *N*-acetylcysteine (NAC) in schizophrenia resistant to clozapine: a double blind randomised placebo controlled trial targeting negative symptoms

**DOI:** 10.1186/s12888-016-1030-3

**Published:** 2016-09-15

**Authors:** Susan L. Rossell, Paul S. Francis, Cherrie Galletly, Anthony Harris, Dan Siskind, Michael Berk, Kiymet Bozaoglu, Frances Dark, Olivia Dean, Dennis Liu, Denny Meyer, Erica Neill, Andrea Phillipou, Jerome Sarris, David J. Castle

**Affiliations:** 1 Centre for Mental Health, Faculty of Health, Arts and Design, Swinburne University of Technology, Melbourne, VIC Australia; 2 St Vincent’s Mental Health Service, St Vincent’s Hospital, Melbourne, VIC Australia; 3 Monash Alfred Psychiatry Research Centre and The Voices Clinic, The Alfred, Melbourne, VIC Australia; 4 Centre for Chemistry and Biotechnology, School of Life & Environmental Sciences, Deakin University, Geelong, VIC Australia; 5 Psychiatry Department, University of Adelaide, Adelaide, South Australia Australia; 6 Northern Adelaide Health Local Network, Adelaide, South Australia Australia; 7 Discipline of Psychiatry, Sydney Medical School, University of Sydney, Sydney, NSW Australia; 8 Brain Dynamics Centre, The Westmead Institute for Medical Research, University of Sydney, Sydney, NSW Australia; 9 School of Medicine, University of Queensland, Brisbane, QLD Australia; 10 IMPACT Strategic Research Centre, Barwon Health, Deakin University, Geelong, VIC Australia; 11 Baker IDI Heart & Diabetes Institute, Melbourne, VIC Australia; 12 Metro South Addiction and Mental Health Service, Brisbane, QLD Australia; 13 Department of Statistics, Data Science and Epidemiology, Swinburne University of Technology, Melbourne, VIC Australia; 14 ARCADIA Mental Health Research Group, Professorial Unit, The Melbourne Clinic, Department of Psychiatry, The University of Melbourne, Melbourne, VIC Australia; 15 Centre for Human Psychopharmacology, Swinburne University of Technology, Melbourne, VIC Australia; 16 Department of Psychiatry, University of Melbourne, Melbourne, VIC Australia

**Keywords:** *N*-Acetylcysteine, Clozapine, Schizophrenia, Negative Symptoms, Cognition, Biomarkers

## Abstract

**Background:**

Clozapine is an effective treatment for a proportion of people with schizophrenia (SZ) who are resistant to the beneficial effects of other antipsychotic drugs. However, anything from 40–60 % of people on clozapine experience residual symptoms even on adequate doses of the medication, and thus could be considered ‘clozapine resistant’. Agents that could work alongside clozapine to improve efficacy whilst not increasing the adverse effect burden are both desired and necessary to improve the lives of individuals with clozapine-resistant SZ. *N*-Acetylcysteine (NAC) is one such possible agent. Previous research from our research group provided promising pilot data suggesting the efficacy of NAC in this patient population. The aim of the study reported here is to expand this work by conducting a large scale clinical trial of NAC in the treatment of clozapine-resistant SZ.

**Methods:**

This study is an investigator initiated, multi-site, randomised, placebo-controlled trial. It aims to include 168 patients with clozapine-resistant SZ, divided into an intervention group (NAC) and a control group (placebo). Participants in the intervention group will receive 2 g daily of NAC. The primary outcome measures will be the negative symptom scores of the Positive and Negative Syndrome Scale (PANSS). Secondary outcome measures will include: changes in quality of life (QoL) as measured by the Lancashire Quality of Life Profile (LQoLP) and cognitive functioning as measured by the total score on the MATRICS. Additionally we will examine peripheral and cortical glutathione (GSH) concentrations as process outcomes.

**Discussion:**

This large scale clinical trial will investigate the efficacy of NAC as an adjunctive medication to clozapine. This trial, if successful, will establish a cheap, safe and easy-to-use agent (NAC) as a ‘go to’ adjunct in patients that are only partly responsive to clozapine.

**Trial registration:**

Australian and New Zealand Clinical Trials Registration Number: Current Randomised Controlled Trial ACTRN12615001273572. The date of registration 23 November 2015.

## Background

### Schizophrenia

Schizophrenia (SZ) is a severe and enduring mental illness, afflicting 1 % of the population. Despite some treatment advances, most notably the discovery of the antipsychotic drugs, many people with SZ continue to suffer substantial disability associated with ongoing distressing symptoms. For example, in Australia (where this trial will take place) the recent Survey of High Impact Psychosis (SHIP) found that 41.3 % of the 1825 participants with a psychotic illness were, despite treatment, experiencing persistent psychotic symptoms [1]. Negative symptoms and cognitive deficits are the most poorly responsive to pharmacological interventions, yet carry the greatest disability in terms of poorer overall personal, social and occupational functioning.

### Clozapine

Clozapine is the most effective antipsychotic drug for treatment–resistant SZ. Due to the significant adverse event profile, it is generally only prescribed to those who have not responded to other antipsychotics and is often considered a ‘last resort’. Clozapine is recommended in all major treatment algorithms for treatment resistant SZ. It is commonly prescribed in Australia, with 22 % of the SHIP sample being on this agent. However, a substantial proportion of people with SZ do not respond adequately to clozapine, with 40–60 % having residual negative and cognitive symptoms despite an adequate trial of clozapine [2] or due intolerable side effects which limit the option of increasing clozapine dose. Attempts to enhance the therapeutic efficacy of clozapine have met with mixed success. A recent review by the Castle (Chief Investigator of this study) outlined a range of augmentation strategies that have been investigated in people who ‘failed’ clozapine. These include other antipsychotics, antidepressants, mood stabilisers, and glutamatergic agents, and although some showed benefits for a proportion of patients, strategies that have been encouraging in small case series have mostly not been confirmed in subsequent more rigorous studies [3]. Agents that could work alongside clozapine to improve efficacy whilst not increasing the side effect burden are essential if we are to improve to lives of clozapine-resistant SZ patients. One such potential agent is *N*-acetylcysteine (NAC), a glutathione (GSH) precursor.

### *N*-Acetylcysteine (NAC)

NAC has been shown to modulate a number of processes involved in psychiatric disorders including neuroinflammation, oxidative stress, and the regulation of glutamate and dopamine neurotransmitter systems. NAC has been trialled as an augmenting agent for a range of disorders including addiction (smoking, cocaine, cannabis and gambling for example), autism, Alzheimer’s, attention deficit hyperactivity disorder (ADHD), and impulse control disorders (nail biting, skin picking, trichotillomania) to name a few [4]. While more high quality trials are needed to confirm the efficacy of NAC in these disorders, at this stage the results are promising.

The study described here is born out of a previous randomised controlled trial of NAC as an adjunctive therapy for SZ specifically [5]. Investigators for this previous trial assessed the efficacy of NAC as an adjunct to treatment as usual in people with SZ; of the 140 enrolled participants, 45 % were on clozapine. Overall, results showed significant improvements on the Clinical Global Impression-Severity Scale (CGI-S; *d* = 0.43; *p* < 0.05) and Positive and Negative Syndrome Scale (PANSS), negative (*d* = 0.52; *p* < 0.05) and general (*d* = 0.46; *p* < 0.05) subscales. NAC was extremely well tolerated with no significant adverse events reported in the NAC group. Additionally, abnormal involuntary movements (a side effect of antipsychotic medication) were reduced during NAC treatment. Secondary analyses using the clozapine patients only [6], showed these benefits were robust for the clozapine patient group. That is, there were significant reductions in PANSS negative scores from baseline to endpoint in the NAC group. Adjusting for baseline score using least square means, the difference between the placebo (*n* = 24) and NAC (*n* = 25) groups was statistically significant (Mdiff = 3.87, SE = 1.84, 95 % CI [0.14, 7.59], *p* < 0.05, d = 0.61). Importantly, Farokhnia et al. [7], in an independent study, also demonstrated similar improvements for NAC for negative symptoms of SZ (as an adjunct to risperidone). Given these findings, and the lack of studies to date that have specifically targeted those prescribed clozapine, the current trial is timely and warranted. Furthermore, the pilot work clearly indicates the feasibility of the trial design.

### Quality of life

A review of the literature shows that people diagnosed with SZ report a lower quality of life (QoL) than the general population [8, 9]. Contributing factors include psychotic symptoms [10], as well as depression and anxiety [11]. Neurocognition has also been linked to QoL with greater cognitive functioning linked to higher objective but lower subjective QoL [12]. Finally, insight has actually been shown to reduce subjective QoL [13].

### Cognition

Cognitive impairment is common in SZ and is thought to play a role in non-response to drug treatment. Indeed, a recent study by de Bartolomeis et al. [14] established that people with treatment resistant SZ had significantly poorer verbal memory and executive function than responders. Additionally, severity of global cognitive impairment was significantly correlated with negative symptom severity in this treatment resistant cohort. There is now substantial evidence from animal studies that NAC improves cognitive function [15], more specifically attention, executive function and memory. There has been very little investigation of the impact of NAC on cognition in human studies. Some early evidence indicates that it may improve specific areas of cognition including memory [16]. Besides being associated with treatment response, cognitive impairment is also associated with poorer social and occupational outcome [17]. Research suggests that improvements in cognition (through cognitive remediation for example) are associated with improvements in these areas [18]. Thus, in the proposed study, we will investigate neurocognitive changes as a secondary outcome measure. We will include premorbid IQ, current IQ and general cognition as examined using the MATRICS, which is considered the gold-standard cognitive assessment in clinical trials for patients with SZ [19].

### Biological mechanisms

Our study has also been designed to enable a novel investigation of the biological effects of NAC treatment. This includes the measurement of peripheral markers including thiols (such as GSH, cysteine, cysteinyl-glycine, NAC) and corresponding disulphides (such as glutathione disulphide (GSSG) and cystine). These analyses will be used to track longitudinal changes in GSH and related compounds through the course of the proposed trial. In addition to peripheral GSH, we will measure cortical GSH via proton magnetic spectroscopy (MRS). Recently there have been substantial developments in both the field strength and spectral acquisition of MRS which now permits the accurate measurement of GSH in the human brain *in vivo*. For example, Do et al. [20] demonstrated that GSH is reduced in the cortex of SZ patients using MRS. Therefore, we will use *in vivo* MRS to assess changes in cortical GSH levels in a subset of participants in the proposed study. This will be completed by obtaining the concentration levels of GSH in the medial prefrontal cortex [21]. The longitudinal changes of these concentrations will be mapped at three time periods across the proposed study: between baseline and 8 weeks to examine for rapid changes; and then at 52 weeks to assess long-term change. Concentrations of GSH across the three time periods will be used in our group-based analyses. The collection of both peripheral and cortical data will allow a full characterisation of the effects of NAC on GSH by exploring links between blood and MRS GSH levels.

### Research aims

This project seeks to expand our existing work by conducting a multi-site randomised placebo-controlled trial of NAC in the treatment of clozapine-resistant SZ, with 8, 24 and 52 week assessments. We will target negative symptoms (primary outcome), quality of life and cognition (secondary outcomes). An additional novel aspect of this proposed study is the examination of both peripheral and cortical GSH concentrations, the latter in a subset of patients using MRS. This will be the first study using NAC to target drug resistant clozapine treated SZ patients specifically; and the first to conduct a 52-week outcome study of the efficacy of NAC in SZ. It is hypothesised that NAC will be superior to placebo in the treatment of negative symptoms in SZ patients currently taking clozapine. Additional benefits are predicted in their quality of life and cognitive functioning. Further analysis of peripheral and cortical GSH concentrations is expected to show increases, whereby increases in cortical GSH will increase cortical functioning.

## Methods

### Study design and plan

**Fig. 1:**
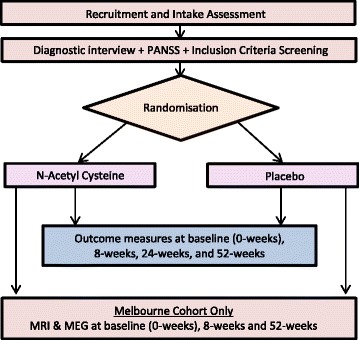
Study Outline

The study is a phase IV, multi-site, 2-arm, 52-week, randomised, double-blind, placebo-controlled trial of 2 g of NAC per day (taken as two 500 mg capsules twice per day or matching placebo), in 168 participants diagnosed with SZ (see Fig. 1 for study outline). In addition to baseline assessments, follow-ups will take place at 8, 24 and 52 weeks (see Table 1 for a list of study assessments). A subsample at the Melbourne site will be invited to undergo a brain scan using MRI (*N* = 42). Scans will be performed at baseline, 8 and 52 weeks. The study protocol was approved by the St Vincent’s Hospital Human Research Ethics Committee (HREC-A HREC-D 030/16), this committee has jurisdiction over all the participating sites. An executive steering committee (all authors) oversees project planning, conduct and ongoing data collation.

**Table 1:** Study Assessments and Timeline

		0 week	2, 4 & 6 w	8 weeks	24 weeks	52 weeks
General	Demographic questionnaire	X		X^1^	X^3^	X^3^
Diagnosis	MINI	X				
Symptoms	Interview to rate PANSS and CAINS	X		X	X	X
	Calgary depression scale for schizophrenia (CDSS) combined with the Montgomery Asberg Depression Scale (MADRS)	X		X	X	X
Safety	Adverse symptoms checklist (SAFTEE)	X	X^2^	X	X	X
	ECG	X				X
	Blood test for clozapine monitoring	X		X	X	X
QoL	Manchester Quality of Life Scale (MANSA)	X		X	X	X
	Assessment of Quality of Life (AQoL)	X		X	X	X
Cognition	The Wechsler test of adult reading (WTAR): Premorbid IQ	X				
	Wechsler abbreviated scale of IQ (WASI): Current IQ	X		X	X	X
	MATRICS	X		X	X	X
Biomarkers	Blood sample for biomarkers	X		X	X	X
Neuro-imaging^3^	Magnetic resonance spectroscopy (MRS)	X		X		X

^1^Shortened version to note for changes in work, study, living arrangements

^2^Telephone safety check

^3^Only participants who are in the Melbourne arm and provide informed consent for the imaging component will be administered these additional assessments

### Setting

The study will be co-ordinated from the Mental Health Department of St Vincent’s Hospital, a large metropolitan teaching hospital in Melbourne, Australia. The study will be conducted across four Australian sites: Melbourne (St Vincent’s Mental Health Service), Sydney (Cumberland Hospital), Adelaide (Northern Adelaide Local Health Network), and Brisbane (Metro South Addiction and Mental Health Service).

### Participants

Forty-two patients will be recruited from each of the sites, with a total target of 168 participants aged 18–65 years meeting DSM-5 criteria for SZ. Investigators at each site will be responsible for recruiting participants through advertising and engagement with clozapine clinics associated with their site. As we are interested in clozapine patients who have residual symptoms, all potential participants will be screened and assessed for eligibility. The following inclusion criteria are to be adhered to: (1) Aged 18–65 years; (2) Confirmed diagnosis of SZ via clinical interview; (3) Have been on clozapine at an adequate dose for at least 6 months, ascertained by a current serum level of >350 mcg/L and with residual symptoms (see 4.)^1^; (4) Have a PANSS score of ≥60 or at least two negative symptom items of >4^2^; (5) Have the capacity to provide consent to the study; and (6) Be utilising effective contraception (i.e., oral or barrier contraception) if female and of childbearing age. In addition, the following exclusion criteria will be applied: (1) Participants who are currently taking NAC; (2) Participants who are allergic to NAC or any component of the preparation; (3) Participants who are currently prescribed nitro-glycerine (significant potential interaction with NAC); (4) Participants who have diabetes mellitus and on insulin replacement (including exenatide) (moderate interaction); (5) Participants taking Aralen (moderate interaction); (6) Participants who regularly take >200 mg/day selenium (moderate interaction); (7) Participants who regularly take anticoagulants (moderate interaction) (excluding aspirin and non-steroidal anti-inflammatories); (8) Change in medication from clozapine to another antipsychotic; (9) Participants with known primary or secondary autoimmune disorders; (10) Recent gastrointestinal ulcers; (11) Recent renal stones; and (12) Females who are pregnant or lactating.

Power was calculated to detect a medium effect size of *d* = 0.5 as described in an earlier NAC and SZ paper [5] and the calculations followed the method described by Diggle et al. [23]. These calculations assumed one primary outcome measure (PANSS Negative scores), four assessment points (baseline, 8-weeks, 24-weeks, and 52-weeks follow-ups), a study-wide Type I error rate (α) of 0.01, a Type II error rate (β) of 0.10 (power of 0.90), a correlation of post-treatment scores with baseline measurements (ρ) of 0.70, employing a two-tailed statistical test. To detect the effect size of *d* = 0.5, 45 participants in each of the control and intervention groups will be required. Allowing for up to 46 % attrition (based on a 20 % dropout over the first 8 weeks and a further 30 % dropout over the ensuing 44 weeks), a total of 168 participants, or 84 in each group will be recruited. Similar attrition rates are reported in other treatment trials with treatment resistant SZ. To accommodate the required number of patients, at least 42 will need to be recruited from each of the four study sites. For the MRS component, groups of 15–20 are widely accepted as an appropriate size to permit appropriate neuroimaging analysis. As noted earlier the anticipated numbers, including drop out and ineligibility for the MRS component are 40 at baseline, 36 at 8-weeks and 52-weeks. Thus, the current study will be adequately powered for the MRS component.

### Consent

The process of consent will be in accordance with the Declaration of Helsinki. All eligible patients will be fully informed that they are being asked to participate in a RCT. The procedures involved in the study, and the chances of being assigned randomly to one of two groups will be explained verbally and via an information sheet approved by the hospital’s Human Research Ethics Committee. All participants will provide informed consent by reading the information sheet, asking any questions they need to and subsequently signing the consent form in front of an independent witness. Participants will be made aware of their right to withdraw from the study at any time without any effects on their clinical management.

### Randomisation and blinding

The study procedure will follow the SPIRIT guideline [24]. The trial is a randomised, controlled, double-blinded superiority trial of two parallel patient groups. Randomisation will be performed independently of the researchers by an independent researcher and will employ block randomisation (2 × 4) with a 1:1 allocation to NAC or placebo. Allocation will be concealed from local investigators, who will assign a number to the participant that will be linked to a prescription from the associated pharmacy. All participants will be dispensed the same number of capsules each month.

### Withdrawal criteria

Participants may be withdrawn from the study for the following reasons: cessation of clozapine; non-adherence to medication; emergence of adverse events; pregnancy/cessation effective contraception; or withdrawal of consent. If a participant is prematurely withdrawn from the study for any reason after product administration, the investigator will make every effort to perform these follow up assessments: review of medications and adverse events. This information will be recorded in the case report form (CRF). In all cases, the reason for withdrawal will be recorded in the CRF.

### Treatment of participants

The trial treatments are NAC and placebo. Participants must take 2 capsules twice per day (total 2 g of NAC) or matched placebo for 52 weeks. NAC is a GSH precursor. It is very efficiently bioavailable and is rapidly deacetylated by the liver into cysteine. Plasma cysteine levels can be rapidly increased by NAC supplementation. NAC rapidly and safely increases plasma cysteine levels, indirectly replenishing depleted systemic GSH. It is available over the counter and is approved in Australia by the Therapeutic Goods Administration (TGA) in injectable and inhalant forms for treatment of paracetamol overdose.

The NAC and placebo capsules will be identical in colour, size and shape, and will be packaged in identical bottles. NAC has a mild odour and to cover this difference between placebo and NAC, a sachet of NAC powder will be placed in all medication bottles so the same smell is present for all study capsules. Each of the treatments will be clearly labelled with a treatment number by an independent researcher. The trial products will be stored in accordance with manufacturer’s instructions which adopt Pharmaceutical Good Manufacturing Practice. Until dispensed to the participants, the trial products will be stored in a securely locked area, accessible only to authorised personnel, in accordance with GCP drug storage requirements.

Participants will be required to take the capsules as instructed during the 52 weeks of the study. At each assessment session they will be asked to return all unused capsules which will be counted for compliance rate. If less than 50 % of the capsules have been taken at any assessment point this will be viewed as non-compliance.

### Assessments

The Mini International Neuropsychiatric Interview – (MINI 6.0) will be used to confirm the diagnosis of SZ and to screen for other co-morbid conditions. The MINI 6.0 is a brief, fully structured instrument which assesses the presence of DSM-5 mood disorders, anxiety disorders, substance use disorders, and antisocial personality disorder. It employs different time frames for various disorders: current or past. The questions asked are aimed at specific psychological problems which require only ‘yes’ or ‘no’ answers. Psychometric evaluation of the MINI 6.0 shows acceptable test-retest and inter-rater reliability [25].

## Outcome and process measures

### Primary outcome measures

#### PANSS and CAINS Symptoms

The PANSS [26] and the Clinical Assessment Interview for Negative Symptoms (CAINS) [27] will be administered at baseline, 8, 24 and 52 weeks. The PANSS assesses Global Positive, Negative and General Psychopathology, and provides a well standardised method of evaluating and monitoring psychotic symptoms. The CAINS provides a more detailed examination of negative symptoms specifically. In the baseline assessment participants will be screened for the presence of negative symptoms with the PANSS and CAINS and only included in the study if they score PANSS >60 total or have at least two negative symptom items of >4.

In addition to these measures, the Calgary Depression Scale for Schizophrenia (CDSS) [28] combined with the Montgomery Asberg Depression Scale (MADRS) [29] will be administered so depression can be accounted for in the analysis of negative symptoms. The diagnostic interview and PANSS/CAINS assessment can take up to 90 min to complete. As previously noted if, after the MINI and PANSS/CAINS interviews are completed the participant is found not to fulfil the inclusion criteria the rest of the clinical interview (general measures and other symptom measures) will not be completed. The primary outcome measure for this trial will be changes to scores on the negative subscale of the PANSS and CAINS. Reductions in severity of negative symptoms in the NAC group compared with the placebo group will indicate that NAC is successfully treating these symptoms.

### Secondary outcome measures

#### Quality of life

The Manchester Quality of Life Scale (MANSA) [30] is a revised and shortened version of the better known Lancashire Quality of Life Profile. It is a structured self-report interview completed with a trained interviewer that contains 16 items to provide objective and subjective ratings of quality of life in people living with SZ. It will be completed alongside the Assessment of Quality of Life (AQoL-4D) [31]. This is a 12-item questionnaire assessing quality of life across four dimensions: independent living, relationships, mental health and sense. A secondary outcome measure for this trial will be changes on these two measures of QoL. Changes indicating improvement in the NAC group specifically will indicate the impact of NAC in the predicted direction.

### Cognition

A comprehensive assessment of neurocognition across multiple domains will be performed. It will include: (1) The Wechsler Test of Adult Reading, WTAR [32], a short (1 min) word reading task that provides a measure of premorbid intelligence; (2) The Weschler abbreviated scale of IQ (WASI) [33], a 10 min assessment of current IQ; and (3) The MATRICS battery [34], an eight-domain cognitive battery that has been constructed to examine the major cognitive impairments reported in psychosis and related conditions. It assesses speed of processing, attention, verbal learning, working memory, visual learning, reasoning and problem solving, and social cognition. The MATRICS takes no longer than 80 min to complete. A scaled score can be calculated for each of the domains along with an overall general cognition score. Another secondary outcome measure of this trial will be cognitive performance. Significant improvements in cognitive performance in the NAC group over the placebo group will indicate the impact of NAC in the predicted direction.

### Process measures

#### Bloods: Peripheral glutathione

Blood samples will be collected. Biomarkers include thiols (such as GSH, cysteine, cysteinyl-glycine, NAC) and corresponding disulphides (such as glutathione disulphide (GSSG) and cystine). This collection for biomarker analyses will occur at baseline, 8, 24 and 52 weeks, to monitor for peripheral changes.

### Magnetic Resonance Spectroscopy (MRS): Cortical glutathione

A sub-set of participants (*N* = 42) will be invited to undergo MRI scans. Scans will be acquired using Swinburne University’s (Melbourne) Siemens Trio 3 Tesla MRI (Siemens AG, Munich, Germany). Participants will spend 1 hour in the scanner. An MRS sequence enables the measurement of levels of metabolites found in the brain. The cerebral metabolites that will be measured in the current study are creatine (Cre) and GSH. MRS voxels will be prescribed in the medial prefrontal cortex (mPFC) and will be 50 × 30 × 30 mm. The voxel will be positioned using an axial T1-weighted gradient-echo image. Spectral processing with be performed using *LCModel* software which permits us to calculate GSH concentrations (calculated in reference to total Cre).

### Safety

The Adverse Symptom Checklist (known as SAFTEE) is a 21-item scale that rates the presence and severity (0 = not present to 3 = severe) of common symptoms associated with psychotropic treatments. As well as being assessed at baseline, 8, 24 and 52 weeks, a telephone assessment at 2, 4 and 6 weeks will also be completed using this assessment. Clozapine levels will be measured at baseline and 8, 24 and 52 weeks to ensure adherence and to ascertain any pharmacokinetic interactions with NAC (we do not expect any such effects as the kinetics of the two agents do not suggest interaction). As well as clozapine levels, hepatic and renal function, full blood count, other metabolic information and electrocardiogram (ECG) will be performed as part of routine clozapine monitoring will be maintained according to local requirements.

## Data management

Data will first be entered into a handwritten patient file and later entered into a computerised database designed for this trial. Double data entry will be completed for 10 % of all study files.

### Statistical analyses

Data will be analysed using R 3.1.2 (R Foundation for Statistical Computing, Vienna, Austria and SPSS where relevant). Intention-to-treat analyses will be employed to prevent over-estimation of efficacy. Categorical variables will be analysed using chi-squared tests (or Fisher’s exact test for small samples). A mixed-effects model, repeated measures (MMRM) approach will be used to examine the longitudinal profile of all continuous variables at weeks 8, 24, and 52 post-baseline. For all MMRM analyses, baseline scores will be used as covariates and the models will include pre-specified fixed effects of treatment, site, and time, and treatment-by-time and treatment-by-site interactions.

The MMRM modelling for the primary outcome measure – PANSS negative scores and CAINS scores – will also include baseline PANSS Positive, Negative, and General Psychopathology scores, as covariates. The secondary outcomes consist of MANSA, AQoL and MCCB global score, while the process outcomes include GSH concentrations peripherally and cortically, respectively.

Secondary analyses using Analysis of Covariance will be conducted to compare change scores during treatment and follow-up phases for all primary, secondary, and process outcomes using treatment group as a main effect with the baseline score as a covariate. Site will not be included in the analysis for the MRS data, but will be for the peripheral data. Correlational analyses will also be performed to examine for relationships between outcome variables (i.e., treatment response with cognition and GSH levels, peripherally and cortically).

Although the attrition rate is not expected to vary by treatment condition, we will attempt to identify key predictors of attrition status (i.e., demographic and baseline clinical characteristics) and test for differences between conditions. Assuming the data are missing at random, several procedures offer effective approaches that may deal with attrition, statistically. Maximum likelihood models, with time as a random variable, allow the use of all available data from all assessments, reducing bias and increasing power [35]. There are three other accepted approaches to analyses with missing data: (a) multiple imputation procedures that utilise the expectation-maximization (EM) algorithm with bootstrap estimates of standard errors; (b) raw maximum-likelihood analysis; or (c) multiple-group structural equation or latent growth modelling [23]. For manifest variable models, these methods produce virtually identical results and will be used to address attrition. In general, the application of these missing data procedures can provide unbiased estimates, even in the face of substantial attrition.

### Data monitoring

A Data Safety Monitoring Board (DSMB) will oversee the conduct of the current trial. This board includes four experts external to the trial team.

## Discussion

SZ is one of the most disabling chronic diseases for people aged 18–45 years, and impacts multiple domains of functioning as well as increasing mortality risks. Current treatments remain suboptimal for many patients, with residual negative and cognitive symptoms being particularly disabling. Improvements and advances in the intervention options for treatment-resistant psychosis are in great demand. Whilst clozapine has profound benefits for some individuals who do not respond to other antipsychotics, it is not as effective for many others. This large scale clinical trial will investigate the efficacy of NAC as a possible adjunctive medication to clozapine. In addition, this trial will run for 52 weeks providing the longest follow up examination of the impact of NAC on SZ to date. This trial, if successful, will establish a cheap, safe and easy-to-use agent (NAC) as a ‘go to’ adjunct in patients only partly responsive to clozapine. This work will provide crucial information regarding the clinical value of this treatment approach. Given the personal and societal cost of treatment resistant psychosis, the research will therefore have great importance for this mental health condition. In addition, we will be able to assess in detail the effects of this agent on cognitive functioning as well as investigating in some detail the glutathione hypothesis of SZ, by examining peripheral and cortical markers of glutamatergic activity.

### Trial status

Patient recruitment was about to commence at the time of manuscript submission. Data collection will continue until at least June 2019.

Australian and New Zealand Clinical Trials Registration Number (TRN) ACTRN12615001273572 https://www.anzctr.org.au/Trial/Registration/TrialReview.aspx?id=369601.

## Abbreviations

AQoL: Assessment of Quality of Life
CAINS: Clinical Assessment Interview for Negative Symptoms
GSH: Glutathione
MANSA: Manchester Quality of Life Scale
MMRM: Mixed-effects model, repeated measures
MRS: Proton magnetic spectroscopy
NAC: *N*-Acetylcysteine
PANSS: Positive and Negative Syndrome Scale
QoL: Quality of life
SHIP: Survey of High Impact Psychosis
SZ: Schizophrenia.

## Footnotes

1 Treatment resistance will be defined using the criteria of Kane et al. [22], viz: at least three periods of treatment in the preceding 5 years with antipsychotic drugs from different chemical classes without significant symptomatic relief: in Australia, these criteria are obligatory in any event for commencement of clozapine.

2 As we are interested in clozapine patients who have residual symptoms, all potential participants will be screened and assessed for eligibility according to these criteria.

## References

[1] Morgan VA, Waterreus A, Jablensky A, Mackinnon A, McGrath JJ, Carr V (2012). People living with psychotic illness in 2010: The second Australian national survey of psychosis. Aust N Z J Psychiatry.

[2] McEvoy JP, Lieberman JA, Stroup TS, Davis SM, Meltzer HY, Rosenheck RA (2006). Effectiveness of clozapine versus olanzapine, quetiapine, and risperidone in patients with chronic schizophrenia who did not respond to prior atypical antipsychotic treatment. Am J Psychiatry.

[3] Castle DJ, Keks N. What Can We Do If Clozapine Fails? Pharmacologic Choices and Differential Outcomes. Treatment–Refractory Schizophrenia. Heidelberg: Springer-Verlag; 2014. p. 93–106.

[4] Deepmala A, Slattery J, Kumar N, Delhey L, Berk M, Dean O, Frye R (2015). Clinical trials of N-acetylcysteine in psychiatry and neurology: A systematic review. Neurosci Biobehav Rev.

[5] Berk M, Copolov D, Dean O, Lu K, Jeavons S, Schapkaitz I (2008). N-acetyl cysteine as a glutathione precursor for schizophrenia—a double-blind, randomized, placebo-controlled trial. Bio Psychiatry.

[6] Dean OM, Mancuso SG, Bush AI, Copolov D, Do KQ, Cuénod M, Rossell SL, Castle DJ, Berk M (2015). Benefits of adjunctive N-acetylcysteine in a sub-group of clozapine-treated individuals diagnosed with schizophrenia. Psychiatry Res.

[7] Farokhnia M, Azarkolah A, Adinehfar F, Khodaie-Ardakani M-R, Yekehtaz H, Tabrizi M (2013). N-acetylcysteine as an adjunct to risperidone for treatment of negative symptoms in patients with chronic schizophrenia: a randomized, double-blind, placebo-controlled study. Clin Neuropharmacol.

[8] Pitkänen A, Hätönen H, Kuosmanen L, Välimäki M (2009). Individual quality of life of people with severe mental disorders. J Psychiatr Mental Health Nurs.

[9] Tan EJ, Thomas N, Rossell SL (2014). Speech disturbances and quality of life in schizophrenia: Differential impacts on functioning and life satisfaction. Comp Psychiatry.

[10] Dan A, Kumar S, Avasthi A, Grover S (2011). A comparative study on quality of life of patients of schizophrenia with and without depression. Psychiatry Res.

[11] Kao Y, Liu Y, Chou M, Cheng T (2011). Subjective quality of life in patients with chronic schizophrenia: relationships between psychosocial and clinical characteristics. Compr Psychiatry.

[12] Kurtz MM, Tolman A (2011). Neurocognition, insight into illness and subjective quality-of-life in schizophrenia: what is their relationship?. Schiz Research.

[13] Ramadan ES, El Dod AW (2010). Relation between insight and quality of life in patients with schizophrenia: role of internalized stigma and depression. Curr Psychiatry.

[14] de Bartolomeis A, Balletta R, Giordano S, Buonaguro EF, Latte G, Iasevoli F (2013). Differential cognitive performances between schizophrenic responders and non-responders to antipsychotics: Correlation with course of the illness, psychopathology, attitude to the treatment and antipsychotics doses. Psychiatry Res.

[15] Cao L, Li L, Zuo Z (2012). N-acetylcysteine reverses existing cognitive impairment and increased oxidative stress in glutamate transporter type 3 deficient mice. Neuroscience.

[16] Adair JC, Knoefel JE, Morgan N (2001). Controlled trial of N-acetylcysteine for patients with probable Alzheimer’s disease. Neurology.

[17] Green MF, Kern RS, Heaton RK (2014). Longitudinal studies of cognition and functional outcome in schizophrenia: implications for MATRICS. Schiz Res.

[18] Wykes T, Newton E, Landau S, Rice C, Thompson N, Frangou S (2007). Cognitive remediation therapy (CRT) for young early onset patients with schizophrenia: An exploratory randomized controlled trial. Schiz Res.

[19] Buchanan RW, Keefe RSE, Umbricht D, Green MF, Laughren T, Marder SR (2011). The FDA-NIMH-MATRICS Guidelines for Clinical Trial Design of Cognitive-Enhancing Drugs: What Do We Know 5 Years Later?. Schiz Bull.

[20] Do K, Trabesinger A, Kirsten‐Krüger M, Lauer C, Dydak U, Hell D (2000). Schizophrenia: glutathione deficit in cerebrospinal fluid and prefrontal cortex *in vivo*. Eur J Neurosci.

[21] Godlewska BR, Yip SW, Near J, Goodwin GM, Cowen PJ (2014). Cortical glutathione levels in young people with bipolar disorder: a pilot study using magnetic resonance spectroscopy. Psychopharmacology.

[22] Kane J, Honigfeld G, Singer J, Meltzer H (1988). Clozapine for the treatment-resistant schizophrenic: a double-blind comparison with chlorpromazine. Arch Gen Psychiatry.

[23] Diggle P, Heagerty P, Liang K-Y, Zeger S. Analysis of longitudinal data. UK: Oxford University Press; 2002.

[24] Chan A-W, Tetzlaff JM, Altman DG, Laupacis A, Gøtzsche PC, Krleža-Jerić K, Hróbjartsson A, Mann H, Dickersin K, Berlin J, Doré C, Parulekar W, Summerskill W, Groves T, Schulz K, Sox H, Rockhold FW, Rennie D, Moher DSPIRIT (2013). Statement: Defining standard protocol items for clinical trials. Ann Intern Med.

[25] Sheehan DV, Lecrubier Y, Sheehan KH, Amorim P, Janavs J, Weiller E (1998). The Mini-International Neuropsychiatric Interview (MINI): the development and validation of a structured diagnostic psychiatric interview for DSM-IV and ICD-10. J Clin Psychiatry.

[26] Kay SR, Flszbein A, Opfer LA (1987). The positive and negative syndrome scale (PANSS) for schizophrenia. Schizo Bull.

[27] Kring AM, Gur RE, Blanchard JJ, Horan WP, Reise SP (2013). The Clinical Assessment Interview for Negative Symptoms (CAINS): final development and validation. Am J Psychiatry.

[28] Addington D, Addington J, Maticka-Tyndale E. Assessing depression in schizophrenia: the Calgary Depression Scale. Brit J Psychiatry. 1993;39–44. 8110442

[29] Montgomery SA, Asberg M (1979). A new depression scale designed to be sensitive to change. Brit J Psychiatry.

[30] Priebe S, Huxley P, Knight S, Evans S (1999). Application and results of the Manchester Short Assessment of Quality of Life (MANSA). Int J Soc Psychiatry.

[31] Richardson J, Hawthorne G (1998). The Australian quality of life (AQoL) instrument: Psychometric properties of the descriptive system and initial validation. Aust Stud Health Serv Adm.

[32] Wechsler Corporation (2001). Wechsler Test of Adult Reading™ (WTAR™).

[33] Wechsler D (1999). Wechsler Abbreviated Scale of Intelligence.

[34] Kern RS, Nuechterlein KH, Green MF, Baade LE, Fenton WS, Gold JM (2008). The MATRICS Consensus Cognitive Battery, part 2: co-norming and standardization. Am J Psychiatry.

[35] Louwerse ES, Weverling GJ, Bossuyt PM, Meyjes FEP, de Jong JV (1995). Randomized, double-blind, controlled trial of acetylcysteine in amyotrophic lateral sclerosis. Arch Neurol.

